# Recombinant Phospholipase D from *Loxosceles gaucho* Binds to Platelets and Promotes Phosphatidylserine Exposure

**DOI:** 10.3390/toxins9060191

**Published:** 2017-06-13

**Authors:** Daniel A. Fukuda, Maria C. Caporrino, Katia C. Barbaro, Maisa S. Della-Casa, Eliana L. Faquim-Mauro, Geraldo S. Magalhaes

**Affiliations:** Laboratory of Immunopathology, Butantan Institute, Av. Vital Brazil 1500, 05503-900 São Paulo, SP, Brazil; dakiofukuda@gmail.com (D.A.F.); maria.caporrino@butantan.gov.br (M.C.C.); katia.barbaro@butantan.gov.br (K.C.B.); maisa.dellacasa@butantan.gov.br (M.S.D.-C.); eliana.faquim@butantan.gov.br (E.L.F.-M.)

**Keywords:** phospholipase D, *Loxosceles gaucho*, EGFP, platelets, binding, chimera toxin

## Abstract

Spider envenomation, from the genus *Loxosceles*, is frequently reported as a cause of necrotic lesions in humans around the world. Among the many components found in the venom of *Loxosceles* genus, phospholipases D (PLDs) are the most investigated, since they can cause a massive inflammatory response, dermonecrosis, hemolysis and platelet aggregation, among other effects. Even though the PLDs induce strong platelet aggregation, there are no studies showing how the PLDs interact with platelets to promote this effect. Since many agonists must interact with specific receptors on the platelet membrane to induce aggregation, it is reasonable to expect that the PLDs may, in some way, also interact with platelets, to induce this activity. Therefore, to address this possibility, in this work, a recombinant PLD, called LgRec1, from *L. gaucho* was fused to enhanced green fluorescent protein (EGFP) and used as a probe to detect the interaction of LgRec1 to platelets, by fluorescence-activated cell sorter (FACS) and confocal microscopy. The preservation of biological activities of this chimera toxin was also analyzed. As a first, the results show that LgRec1 does not require plasma components to bind to platelets, although these components are necessary to LgRec1 to induce platelet aggregation. Also, the attachment of LgRec1 to human platelets’ cell membranes suggests that the exposure of phosphatidylserine (PS) may act as a scaffold for coagulation factors. Therefore, the results add new information about the binding of *Loxosceles* PLDs to platelets, which may help unravel how these toxins promote platelet aggregation.

## 1. Introduction

Envenomation by spiders of the *Loxosceles* genus, also known as brown spiders, can cause significant clinical manifestations in humans, even causing fatalities in some cases [1]. In Brazil, *Loxosceles intermedia*, *Loxosceles laeta*, and *Loxosceles gaucho* spiders are the most important species causing the highest number of incidents [2,3], with thousands of cases being reported annually [4]. To better understand the venom composition, many of its components have been identified, including serine-proteases [5], hyaluronidases [6,7], insecticidal toxins [8], metalloproteases [5,9] and phospholipases D [10,11,12], among others. 

The transcriptome analysis of *L. intermedia* [13] and *L. laeta* [14], allowed for the construction of a better profile of the toxins transcripts present in the venom glands of these spiders, as well as their relative abundance. Among them, the phospholipases D (PLDs), also called dermonecrotic toxins, sphingomyelinases D or SMases, were identified as the most abundant, with many isoforms that may be involved in the adaptation of the spiders, and the effectiveness of the venom. It has been shown that the PLDs can reproduce most of the effects ascribed to the whole venom, including dermonecrosis [11,12], edema [15], nephrotoxicity [16], massive inflammatory response with neutrophil infiltration [17,18], hemolysis [19,20,21] and platelet aggregation [22,23,24,25,26]. Although considerable effort has been made to understand how the PLDs perform their activities, the task is hampered by the several steps required for their purification, and the small amount of venom extracted from the spiders. To overcome this problem, many of these toxins have been cloned and expressed in the bacterial system [27]. In this regard, our group also cloned and characterized the first PLD from *L. gaucho*, called LgRec1. This toxin showed the main biological activities of the venom, such as dermonecrosis, hemolysis and platelet aggregation [26]. 

The use of recombinant PLDs in several studies has allowed for a better understanding of their mechanism of action. In this regard, many works indicate that these toxins can release bioactive metabolites from the lipids present in the cell membrane that ultimately would promote the biological activities [28,29]. In fact, it has been shown that *Loxosceles* PLDs hydrolyze sphingomyelin (SM), generating ceramide 1-phosphate (C1P) or lysophosphatidylcholine (LPC), that once converted to lysophosphatidic acid (LPA), can trigger inflammatory responses [30,31]. Thus, it seems that the PLDs must establish some interaction with the lipids or proteins on the cell membrane in order to exert their action. For instance, the binding of PLDs from *L. intermedia* on B16-F10 murine melanoma [18] and rat aortic endothelial cells [32], has shown to cause the release of choline, a product generated by the action of PLD on sphingomyelin. In addition, the interaction of PLDs with erythrocytes has been extensively studied due to the prominent hemolysis caused by these toxins. In this case, two different mechanisms of interactions with the erythrocytes have been proposed to explain the hemolytic effect [19,20,28,33]. 

Along with erythrocytes, platelets represent another cellular target for the PLDs, since these toxins can induce remarkable platelet aggregation [22,25,26,34,35,36]. This event is related to thrombocytopenia and thrombosis observed in the envenoming [37] that may aggravate the dermonecrosis caused by the PLDs [38]. Although it is hypothesized that platelet aggregation is a result of bioactive lipids released by the action of the PLDs [29], there is some indication that plasma components are needed for platelet aggregation [23,25,26,34]. Hence, the role of PLDs in platelet aggregation still demands an investigation.

In this study, to better understand the relationship between the PLDs and platelets, we fused the recombinant PLD LgRec1 from *L. gaucho*, with enhanced green fluorescent protein (EGFP). This chimeric toxin, called EGFP-LgRec1, was then used as a fluorescence detector. Our results indicated that the fusion of EGFP to LgRec1 did not show interference with the main biological activities of this toxin, such as platelet aggregation, hemolytic and sphingomyelinase activities. In addition, this chimera allowed us to demonstrate and quantify the binding of LgRec1 to platelets through methodologies such as fluorescence activated cell sorter (FACS) and confocal microscopy. Therefore, this work is the first evidence that a *Loxosceles* PLD binds to platelets, triggering the exposure of phosphatidylserine (PS), which consequently promotes their aggregation.

## 2. Results

### 2.1. Cloning, Expression, and Purification of Phospholipase D (LgRec1) Fused to EGFP

**Figure 1:**
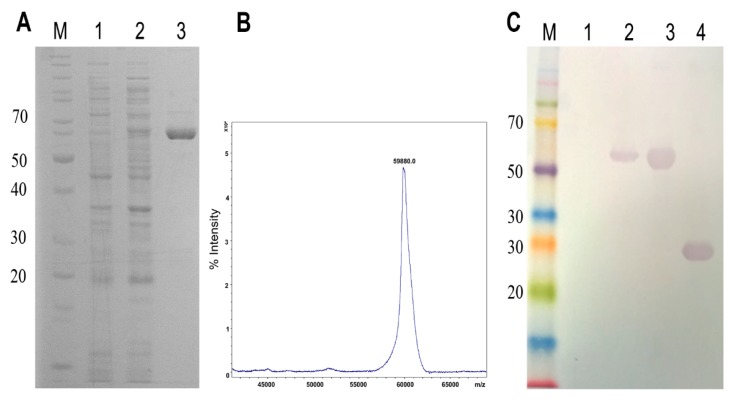
Expression analysis of the chimeric toxin EGFP-LgRec1 (**A**) SDS-PAGE of recombinant EGFP-LgRec1 expression. EGFP-LgRec1 was overexpressed in *E. coli* BL21 Star (DE3) using pAZ vector and IPTG induction (1 mM) overnight at 20 °C. Proteins were visualized on a 12.5% SDS/polyacrylamide gel under reduction conditions and stained with Coomassie blue. (**B**) MALDI-TOF MS analysis of purified EGFP-LgRec1. The spectra were acquired in positive ion linear mode, using the default calibration of the instrument. (**C**) Western blot analysis. Proteins were separated by SDS-PAGE, transferred onto nitrocellulose membrane, and incubated individually with anti-LgRec1 serum. M: protein molecular weight markers; 1 and 2: extract from BL21 Star™ (DE3) cells without and with IPTG induction, respectively; 3: purified/dialyzed EGFP-LgRec1; 4: purified/dialyzed LgRec1.

The sequence of LgRec1 was cloned at a 3′portion of the EGFP sequence in such a manner that after expression, LgRec1 would be located at the C-terminus of EGFP. The construction was transformed into BL21 Star™ (DE3) chemically competent cells, and after expression, the soluble fraction of cell lysates was subjected to Ni^2+^ affinity chromatography and analyzed by SDS-PAGE. A single band around 60 kDa was observed in the purified fraction (Figure 1A). The mass spectrometry analysis showed that the chimeric protein, named EGFP-LgRec1, presented a molecular mass of 59.880 Da (Figure 1B), which correspond to the mass of EGFP with 6xHis tag (28.3 kDa) and LgRec1 (31.7 kDa). Further analysis by western blot using anti-LgRec1 polyclonal antibodies showed the recognition of a single band for the induced and purified EGFP-LgRec1, as well as for the purified recombinant LgRec1 that was used as a positive control (Figure 1C). The average yield of soluble EGFP-LgRec1 was 20 mg per liter of culture.

### 2.2. Biological Characterization of the Chimeric Toxin EGFP-LgRec1

**Figure 2:**
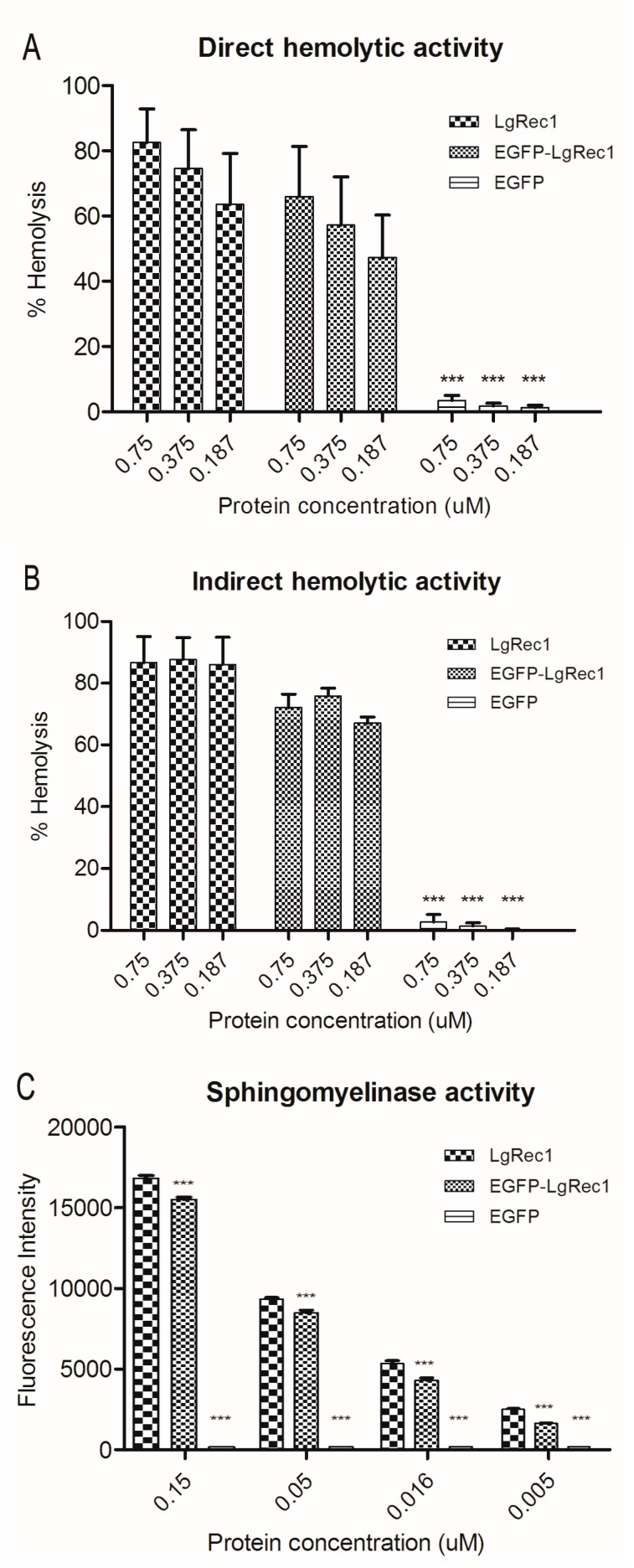
Biological characterization of the recombinant chimeric toxin EGFP-LgRec1. Comparative direct (**A**) and indirect (**B**) hemolytic activity induced by LgRec1, EGFP or EGFP-LgRec1. Indirect hemolytic activity was evaluated in washed erythrocytes after 4 h in the presence of human serum, while direct hemolytic activity was evaluated after 12 h in the absence of serum with 1 mM of Ca^2+^. In both situations, erythrocytes were incubated with different proteins concentration (0.75, 0.375 and 0.187 µM). Values given are the mean ± SEM (*n* = 6), *** *p* < 0.001 vs. LgRec1. (**C**) Comparative sphingomyelinase activity of EGFP-LgRec1, LgRec1 and EGFP evaluated by Amplex-Red Sphingomyelinase Assay Kit at 37 °C for 30 min and fluorescence intensity was measured in a microplate reader using excitation at 540 nm and emission at 590 nm. Reactions used 0.15, 0.05, 0.0016 and 0.005 µM of each recombinant protein. Results were expressed as mean ± SEM (*n* = 3), *** *p* < 0.001 vs. LgRec1.

Since LgRec1 can induce hemolysis and sphingomyelin hydrolysis [26], it was investigated if these activities were not impaired due to its fusion to the EGFP. It is known that the PLDs can induce hemolysis in the presence [19] or absence of serum [20], which we called indirect and direct hemolysis, respectively. Therefore, both situations were tested for EGFP-LgRec1, LgRec1, and EGFP (used as a negative control). As the results show, when tested in the presence of serum (Figure 2A), both toxins could promote hemolysis in 4 h, while in its absence, the hemolysis was prominent only after 12 h (Figure 2B). In each situation, both EGFP-LgRec1 and LgRec1 exhibited similar results. The sphingomyelinase activity, evaluated with the Amplex-Red Sphingomyelinase Assay Kit, also showed that both EGFP-LgRec1 and LgRec1 displayed similar concentration-dependent activity. Taken together, the results indicate that EGFP fusion did not interfere with the main biological activities of LgRec1. 

### 2.3. EGFP-LgRec1 Promotes Platelet Aggregation and Binding in Human Platelet-Rich Plasma 

**Figure 3:**
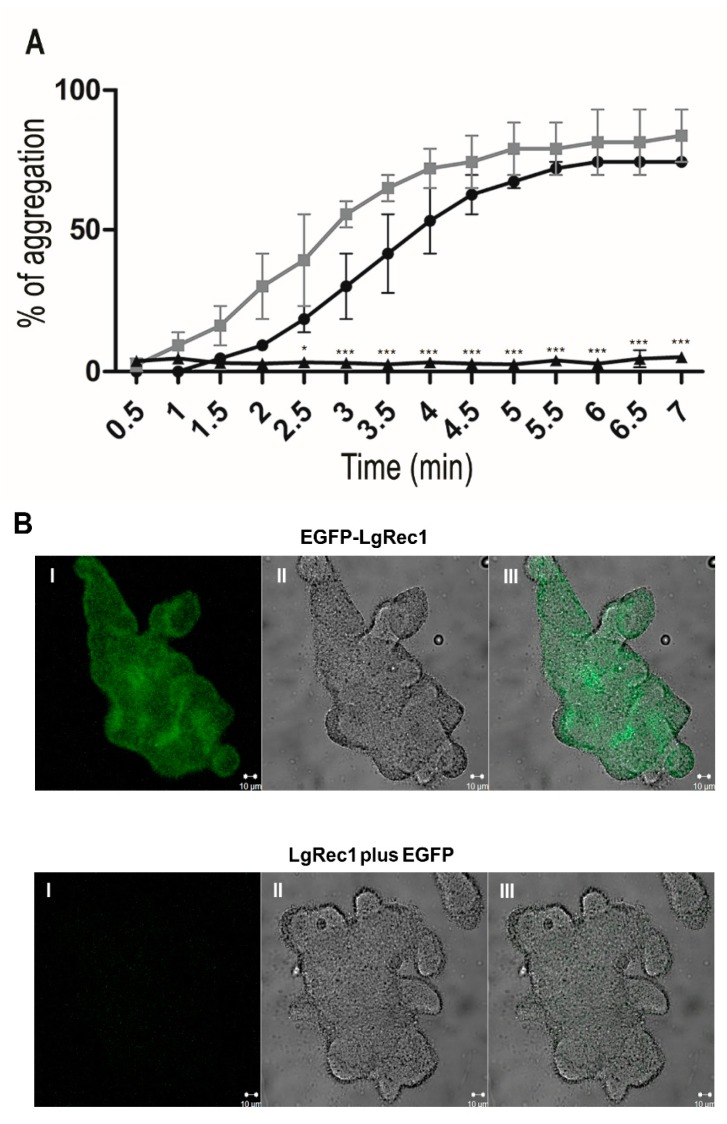
Platelet aggregation of human platelet rich plasma (PRP) treated with the recombinant EGFP-LgRec1 and confocal microscopy images analysis. (**A**) Platelet aggregation assay. Comparative platelet aggregation induced by EGFP-LgRec1 and LgRec1. Platelet-rich plasma was incubated with 0.3 µM of LgRec1, EGFP-LgRec1, and EGFP (negative control), respectively. Aggregation was monitored by measuring the light transmittance for 7 min by an aggregometer. Values given are the mean ± SEM (*n* = 3) * *p* < 0.05 and *** *p* < 0.001 vs. LgRec1. (**B**) Confocal microscopy images analysis. PRP was incubated with 4 µM of the chimeric toxin EGFP-LgRec1 or with LgRec1 plus EGFP for 2 h at 37 °C and analyzed by confocal microscopy. (**I**) fluorescence image (**II**) differential interference contrast image, (**III**) overlay of the left and middle images. Scale bars (10 μM) are shown at the bottom right of the images.

One remarkable characteristic of LgRec1 is its ability to promote platelet aggregation in vitro when incubated with human platelet-rich plasma (PRP) [26]. In this study, LgRec1, EGFP-LgRec1, or EGFP were incubated with human PRP, and their ability to promote platelet aggregation was evaluated. The results showed that both LgRec1 and EGFP-LgRec1 exhibited maximal platelet aggregation after 6 min (Figure 3A). Next, it was analyzed if the aggregation promoted by EGFP-LgRec1 could be related to its binding to the platelets. For this, the platelet aggregate clumps were extensively washed and analyzed by confocal microscopy. Since LgRec1 could cause platelet membrane modifications that would allow for unspecific binding of the EGFP, the LgRec1 plus EGFP in equimolar concentration was used as a control. In this experiment, the EGFP was not used as a control, because it does not induce platelet aggregation, as previously observed. As shown in Figure 3B, while EGFP-LgRec1 could induce platelet aggregation and show fluorescence, LgRec1 plus EGFP only caused platelet aggregation. These results show that LgRec1 binds to platelets and promotes their aggregation in the presence of plasma components.

### 2.4. EGFP-LgRec1 Binds to Platelets in the Absence of Plasma Components

**Figure 4:**
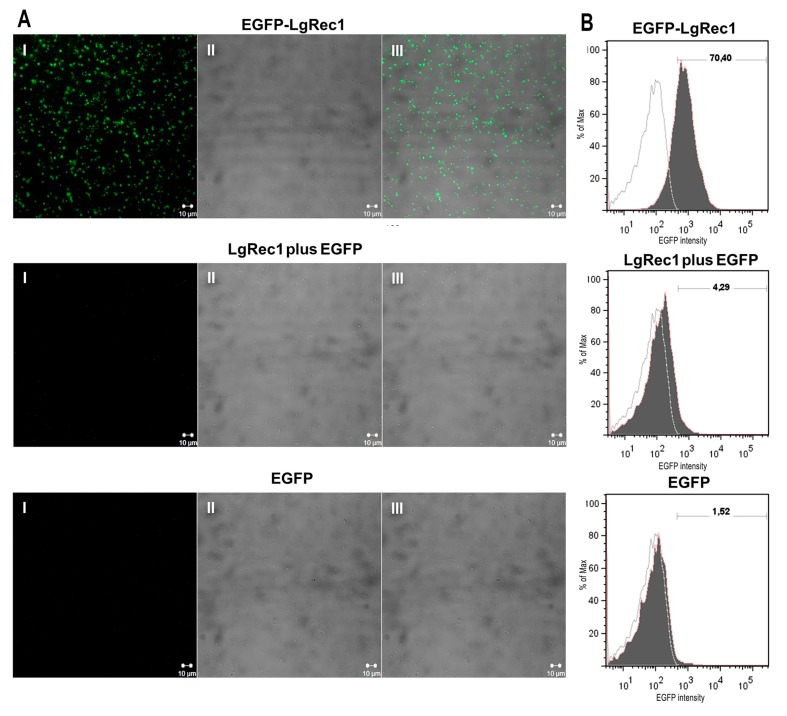
Binding of recombinant EGFP-LgRec1 to human washed platelets. (**A**) Laser scanning confocal microscopy images of fixed human washed platelets. Washed platelets were fixed and incubated with 4 µM of the chimeric toxin EGFP-LgRec1, LgRec1 plus EGFP or EGFP for 2 h at 37 °C. (**I**) fluorescence image (**II**) differential interference contrast (DIC) image, (**III**) overlay of the left and middle images. Scale bars (10 μM) are shown at the bottom right of the images. (**B**) Flow cytometry analysis of washed platelets. Washed platelets incubated with 4 µM of EGFP-LgRec1, LgRec1plus EGFP or EGFP for 2 h at 37 °C. Positive labeled cells were defined as those with a fluorescence intensity above of the untreated platelets. The filled histograms represent the cells labeled with EGFP-LgRec1, LgRec1 plus EGFP or EGFP; the empty histograms represent the same cell suspension untreated. Percentages of labeled platelets after treatment are indicated on the horizontal bar. The samples were performed in duplicate and are representative of three different experiments.

Previous studies have shown that LgRec1 [26] and other *Loxosceles* phospholipases D cannot cause platelet aggregation if plasma components are absent [23,24,25]. For this reason, it was investigated if the binding of EGFP-LgRec1 to the platelets could be affected by the lack of these components. To this end, human platelets were washed, fixed, and treated with the chimera EGFP-LgRec1, LgRec1 plus EGFP, and EGFP. As shown in Figure 4A, the washed platelets treated with EGFP-LgRec1 showed fluorescence, while those treated with both controls did not. To quantify the percentage of washed platelets labeled with EGFP-LgRec1, they were submitted to flow cytometry analysis (Figure 4B). The results showed that EGFP-LgRec1 bound to ~70% of the washed platelets. Thus, these results show that LgRec1 does not require plasma components to bind to platelets, although these elements are necessary to LgRec1 induce platelet aggregation. 

### 2.5. EGFP-LgRec1 Induce Exposure of Annexin-V Binding Sites in Platelets

**Figure 5:**
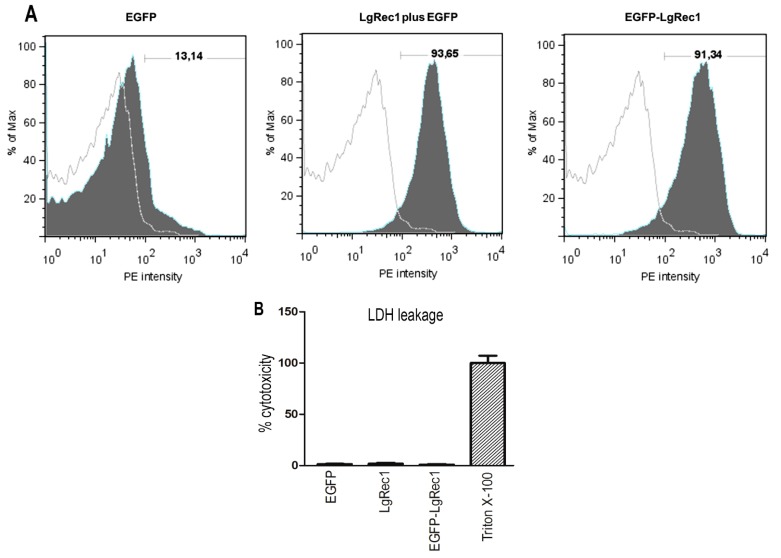
Binding of annexin-V to platelets treated with EGFP-LgREc1 or LgRec1 and its effect on lactate dehydrogenase (LDH) leakage. (**A**) Flow cytometry analysis of platelets. Platelets were treated with 4 µM of EGFP, LgRec1 plus EGFP or the chimeric toxin EGFP-LgRec1 for 2 h and 45 min at 37 °C, then incubated for 15 min at room temperature (25 °C) in the dark with annexin V. Platelets were separated from debris based on the forward- and side-scatter profiles (data not shown). Percentage of labeled platelets obtained after treatment are indicated by horizontal bars; staining positive was defined as those with a fluorescence intensity above that of the untreated platelets. The filled and empty histograms represent treated and untreated cells, respectively. The example is representative of three different experiments, each (*n* = 2). (**B**) LDH release by washed platelets. Washed platelets were incubated with 4 µM of EGFP, LgRec1 or EGFP-LgRec1 and LDH leakage was evaluated by LDH Cytotoxicity Assay Kit (Cayman Chemical, Ann Arbor, MI, USA) at 37 °C for 30 min, and absorbance was measured at 490 nm. Basal levels were assayed in untreated washed platelets, and Triton X-100 was used as maximum cytotoxicity control. Data are expressed as mean ± SEM (*n* = 3).

There are many pieces of evidence indicating that erythrocytes treated with PLDs promoted the exposure of phosphatidylserine (PS) on the surface of these cells [20,33]. Considering that when platelets are activated, PS is also exposed on the cell surface to form a scaffold for coagulation factors [39], we investigated the activity of LgRec1 on the lipid reorganization on the platelet membrane. For this purpose, washed platelets were treated with recombinant toxin and incubated with annexin-V, which binds in a calcium-dependent and reversible manner to PS-expressing membranes [40]. As indicated by Figure 5A, about 90% of platelets were positive for annexin-V when preincubated with LgRec1 or LgRec1 plus EGFP, while EGFP showed only a minor binding, probably due to platelet manipulation during the washing process. Hence, this finding suggests that LgRec1 promotes a reorganization of lipid components on platelet membranes with the exposure of PS. Also, as the process of PS flipping from the inner to the outer leaflet of the platelet membrane is present in cells undergoing apoptosis [39], we evaluated if LgRec1 or EGFP-LgREc1 promote damage to the platelets. To this end, we measured the level of lactate dehydrogenase (LDH), which is released when plasmatic membrane damage occurs. The results indicated similar levels of LDH in the supernatant of platelets incubated with the toxins or EGFP, showing that LgRec1 does not cause platelet lysis during the time that this toxin induces platelet aggregation (approximately 10 min).

## 3. Discussion

The *Loxosceles* PLDs comprise a family of toxins that have amino acids and biological similarities [13,41], and studies using purified recombinant PLDs isoforms from different *Loxosceles* species have shown that they can promote dermonecrosis, deregulated inflammatory responses, hemolysis, platelet aggregation, increased vascular permeability, and acute renal failure [15,16,20,25,26]. Despite the indication that many of these biological activities are somehow related to the phospholipid metabolites generated by the PLDs through their action on the cell membrane, the mechanism of action of these toxins is not yet completely understood. For instance, it has been shown that the lysis of erythrocytes induced by a PLD from *L. laeta* venom is dependent on the activation of the complement system by the alternative pathway [10], which is triggered by the cleavage of cell surface glycophorins by an unknown endogenous membrane-bound metalloproteinase [19,33]. On the other hand, some studies support a direct involvement of a PLD from *L. intermedia* in the hemolytic activity. In this case, the authors postulate that the PLD hydrolyzes sphingomyelin from the erythrocytes membrane, generating ceramide 1-phosphate, that in turn, would stimulate structural changes to the cytoplasmic membrane, causing Ca^2+^ influx and, consequently, hemolysis [20,28]. Thus, it is reasonable to think that these two events together may represent a cooperation to increase the efficiency of these toxins.

Although the mechanism of *Loxosceles* PLDs on hemolysis are being studied in more detail, so far, no studies were conducted to identify the interaction of these toxins with platelets, even though they promote strong platelet aggregation in vitro and thrombus formation in rabbits [25]. Additionally, studies have also suggested a correlation between the depletion of platelets, and the dermonecrotic lesion development induced by *L. gaucho* venom [38] and thrombocytopenia in humans [2,42]. So, in this report, we explored the possibility of using a recombinant PLD LgRec1 from *L. gaucho* [26] fused to the EGFP as a probe to detect the interaction of LgRec1 to platelets. The EGFP was chosen because its single excitation peak at 510 nm adapts perfectly to standard fluorescein isothiocyanate (FITC) filter sets, which makes it ideal to be used as an auto-fluorescent marker in the fluorescence activated cell sorter (FACS) and in the laser scanning confocal microscope (LSCM) [43]. Therefore, using these technologies, the interaction of LgRec1 with cells could easily and rapidly be analyzed. 

To perform the construction of the chimera toxin, the LgRec1 was fused at the C-terminus of EGFP, because this portion is flexible and rarely requires a linker to provide flexibility between the EGFP and the protein of interest [44]. The EGFP-LgRec1 was successfully expressed in bacteria, and mass spectrometry analysis showed the right mass for this construction, indicating that no premature termination occurred. In line with previous studies on PLDs, both LgRec1 and EGFP-LgRec1 showed a faster hemolytic activity in the presence of serum when compared without it, indicating the importance of serum components in the erythrocytes hemolysis [19]. In addition, EGFP-LgRec1 exhibited preserved sphingomyelinase and platelet aggregation activities, showing that the relatively large EGFP (~27 kDa) did not interfere with the main biological activities of LgRec1. 

The first clinical and experimental studies on platelets using *Loxosceles* PLDs was performed by Denny et al. in 1964 [45], suggesting that toxins present in the *L. reclusa* venom could promote platelet activation. Later, in 1981 another group of researchers [22] using a purified PLD from this venom, demonstrated a non-lytic effect on platelets with aggregation and serotonin release. Subsequently, using platelets separated from plasma proteins, Rees et al. in 1988 [34] showed, for the first time, that a purified PLD from *L. reclusa* required a plasma component(s) to promote platelet aggregation. In that study, the plasma component, C-reactive protein (CRP), was seen as a co-factor required for platelet aggregation. However, in another study, the same group established that serum amyloid P component (SAP), not CRP, is most likely the protein required by the toxin to activate human platelets in vitro. The authors claimed that the previous results observed with CRP were probably due to contamination with SAP [23,46]. However, so far, no other study has confirmed this observation, although it has been shown that PLDs require plasma components to induce platelet aggregation [25,26]. Another possible explanation for platelet aggregation caused by PLDs is that these toxins can produce lysophosphatidic acid (LPA) [30,31], which is capable of inducing platelet activation and aggregation through the stimulation of LPA_1_ and LPA_3_ receptors [47]. However, experiments using dioctanoylglycerol pyrophosphate, a selective antagonist of LPA_1_ and LPA_3_ receptors, failed to prevent platelet aggregation induced by *L. gaucho* whole venom [25]. Consequently, the mechanism by which PLDs induce platelet aggregation requires more investigation.

As the EGFP-LgRec1 showed itself to be fully active, it was tested to see if it could induce platelet aggregation in human platelet-rich plasma (PRP), and if this activity was related to the binding of LgRec1 to its membrane. The results showed that EGFP-LgRec1 promoted platelet aggregation in human PRP in a similar way to LgRec1, and the aggregates treated with EGFP-LgRec1 exhibited a strong fluorescence, with no signal detected in the controls. Also, to investigate if EGFP-LgRec1 could bind to platelets in the absence of plasma components, the EGFP-LgRec1 was incubated with washed platelets. The confocal analysis showed that only platelets treated with EGFP-LgRec1 exhibited fluorescence, indicating that although LgRec1 requires plasma components to promote platelet aggregation, these components are not necessary for the binding of this toxin. Further analysis by FACS, revealed that about 70% of the platelets were tagged by EGFP-LgRec1. 

Previous experiments with the PLDs from *L. laeta* [33] and *L. intermedia* [20] have demonstrated the exposure of phosphatidylserine (PS) on the erythrocytes membrane after toxin treatment. This event was associated with the formation of ceramide resulting from PLDs activity on the sphingomyelin present on the erythrocyte membrane [20], since the increment of ceramide levels, along with increased cytosolic Ca^2+^, can lead to membrane scrambling and subsequent PS exposure [48,49]. In platelets, PS exposure leads to a procoagulant phenotype [50], a process also regulated by intracellular Ca^2+^, suggesting the involvement of calcium-activated phospholipid scramblases [39,51]. 

The flow cytometry analysis showed that washed platelets treated with the PLD LgRec1 were positive for the binding of annexin-V, indicating the presence of PS. Our results also indicated that this flipping of PS on platelets caused by LgRec1 did not promote damage to the platelets membrane in the time necessary for this toxin to induce platelet aggregation, since no leakage of LDH was detected. As PS exposure on the surface of activated platelets is essential for the membrane assembly of coagulation factor complexes necessary for thrombin generation and fibrin formation [52], this might explain the induction of platelet aggregation by PLDs in the presence of plasma components. However, whether or not the PS flipping to the outer leaflet of the platelet membrane is caused by ceramide, due to the action of LgRec1, and if this exposure alone is sufficient to support platelet aggregation, independent of other events, such as the release of α-granules, remains to be elucidated. In the future, the determination of the LgRec1 receptor will be an important step to understanding this toxin’s mechanism, which could contribute to the development of inhibitors to prevent platelet aggregation. In this regard, the use of the chimera toxin developed in this work may represent a useful tool for these studies. 

## 4. Conclusions

In this work, we describe the use of EGFP fused to the recombinant PLD LgRec1 from *L. gaucho* venom as a probe to show the first evidence of attachment of this toxin to platelets. The data indicates, that while this toxin requires plasma components to promote platelet aggregation, these components are not necessary for the binding of LgRec1 to platelets. In addition, the attachment of LgRec1 to human platelets cell membrane suggests the exposure of PS that may act as a scaffold for coagulation factors, thus explaining the requirement of plasma components to promote platelet aggregation.

## 5. Materials and Methods

### 5.1. Ethics

All manipulation of microorganisms was approved by CTNBio (n° 2949/2011). All procedures involving human blood were approved by São Paulo Health Institute—Ethical Committee for Human Protocol: 019/2008/SES/IS/CEPIS.

### 5.2. Cloning, Expression, and Purification of EGFP-LgRec1 

To construct the chimera EGFP-LgRec1, the sequence of LgRec1 was extracted from pAE-LgRec1 vector [26] with the help of restriction enzymes *Bam*HI and *Hind*III, then subcloned into the pAZ vector, which contains an EGFP sequence [53], previously digested with the same restriction enzymes. The sequence of EGFP was positioned at the 5′portion of LgRec1, and the resulting vector was named pAZ-EGFP-LgRec1. 

Chemically competent *E. coli* BL21 Star™ (DE3) pLysS (Thermo Fisher Scientific Scientific, Waltham, MA, USA) cells were transformed with pAZ-EGFP-LgRec1 construction and plated on LB-agarose plates containing 100 µg/mL ampicillin and 34 mg/mL chloramphenicol and grown overnight at 37 °C. The next day, a single colony was then inoculated into LB broth (containing 100 µg/mL ampicillin and 34 mg/mL chloramphenicol) and grown overnight at 30 °C. The overnight culture was then used to inoculate 250 mL of fresh LB (ampicillin) medium at 1:50 dilution. At an optical density (600 nm) of 0.6, isopropyl-β-d-1-thiogalactopyranoside (IPTG) was added to a final concentration of 1 mM to induce the recombinant protein expression. The culture was maintained overnight at 20 °C before the cells were harvested by centrifugation at 5000 *g* for 10 min at 4 °C. Cells were re-suspended in binding buffer (20 mM sodium phosphate, 500 mM NaCl, pH 7.0) and intermittently sonicated. Cell debris was removed from the protein solution by centrifugation at 10,000 *g* for 10 min. 

The supernatant containing the soluble proteins was purified by immobilized metal affinity chromatography (IMAC) using Ni^2+^ Sepharose^®^ 6 Fast Flow GE^®^ (GE Healthcare, Little Chalfont, UK) following the manufacturer’s recommendations. The protein was dialyzed against TBS-Glycerol (20 mM Tris, 100 mM NaCl, 1% Glycerol, pH 7.4). Quantification of recombinant toxins was performed using the BCA Pierce™ Protein Assay Kit (Thermo Fisher Scientific, Waltham, MA, USA) following the manufacturer’s protocol. The LgRec1 and EGFP were expressed and purified as previously described [26,53].

### 5.3. Determination of Molecular Mass by Mass Spectrometry

This procedure was carried out by the Center for Research Support (CEFAP) of the Institute of Biomedical Sciences (ICB) of the University of São Paulo (USP). Analyses were performed on the MALDI-TOF Autoflex Speed (Bruker Corporation, Billerica, MA, USA) equipment following pre-established protocols for protein analysis. The procedure consisted in the application of 0.5 μL of a saturated solution of sinapinic acid in ethanol, on the sample of EGFP-LgRec1. After drying, 1 μL of EGFP-LgRec1 was mixed with 1 μL of a TA30 saturated solution (0.1% trifluoroacetic acid/acetonitrile, 70:30) and finally, 1 μL was applied to the Ground Steel plate for analysis. Data acquisition was performed in linear mode with positive polarity, with the following parameters: Ion Source 1—19.50 kV, Ion Source 2—17.60 kV, Lens—9.0 kV, Pulsed Ion Extraction 170 ns, Mass Range 5–70 kDa, Laser Frequency 500 Hz, Gain Detector 10.0×. The results were analyzed using the online software mMass version 5.5.0, 2013 (Martin Strohalm^©^ Open Source Mass Spectrometry Tool) [54]. 

### 5.4. SDS-Polyacrylamide Gel Electrophoresis and Western Blot Analysis 

Samples of the recombinant proteins or bacterial extract culture before and after IPTG induction were analyzed by SDS-PAGE 12.5% under reducing conditions (2.5% dithiothreitol). After electrophoresis, proteins were stained with Coomassie Blue R-250 or transferred onto nitrocellulose membranes using the Trans-Blot^®^ turbo^TM^ transfer system (BioRad^®^, Hercules, CA, USA) following the manufacturer’s recommendations. After transfer, the nitrocellulose membranes were stained with Ponceau S (Merck Millipore Corporation, Darmstadt, Germany) at 1:20 dilution to verify the transfer efficiency. To remove the dye, the membranes were washed with TBS-Tween (20 mM Tris, 150 mM NaCl, 0.05% Tween 20, pH 7.5) until complete removal. The membranes were incubated for 2 h with rabbit polyclonal anti-LgRec1 antibody at 1:8000 dilution. Membranes were washed with TBS-Tween and immunoreactive proteins were detected using 1:1000 diluted peroxidase labeled anti-rabbit IgG (Sigma Life Science, St. Louis, MO, USA), and the blot revealed with 4-chloro-1-naphthol. To infer the molecular mass, the following standards were used: Full-range Amersham Rainbow Marker (GE Healthcare, Little Chalfont, UK) and PageRuler™ Prestained Protein Ladder (Thermo Fisher Scientific, Waltham, MA, USA).

### 5.5. Sphingomyelinase Assay

Sphingomyelinase activity was measured by Amplex^®^ Red Sphingomyelinase Assay Kit (Thermo Fisher Scientific, Waltham, MA, USA). This assay specifically monitors PLD activity by using choline oxidase to oxidize free choline released after cleavage of the phosphodiester bond of sphingomyelin. Released hydrogen peroxide binds to AmplexTM Red resulting in a fluorescent molecule (resorufin). The chimeric toxin EGFP-LgRec1, LgRec1 with EGFP or EGFP (0.15, 0.05, 0.016 and 0.005 µM) were added to the Amplex-Red reagent mixture and the plate containing the samples (100 µL) were incubated at 37 °C for 30 min before measuring fluorescence absorbance/emission at 540/590 nm in a spectrofluorometer Spectramax M2 (Molecular Devices LLC., Sunnyvale, CA, USA).

### 5.6. Blood Cells and Serum 

Human blood was collected in a 3.8% sodium citrate solution (1:9), after informed consent, from healthy volunteers who declared not to have taken medications known to interfere with platelet function for the previous 10 days. Further steps were taken depending on the wanted cell, as following:

Platelet Rich Plasma (PRP): Citrated whole blood was centrifuged at 115 *g* for 30 min, and the supernatant was collected.

Washed platelets: Prostaglandin E1 (PGE1) was added to PRP and centrifuged at 1125 *g* for 15 min and the pellet was washed twice with wash buffer (60 mM NaCl, 2.7 mM NaHCO_3_, 1.27 mM KCl, 0.45 mM Na_2_HPO_4_, 1.05 mM MgCl_2_. 10.98 mM Na_3_Citrate, 0.27 mM glucose, 26.33 mM BSA, pH 6.5). The washed pellet was then resuspended at Tyrode’s solution (66.9 mM NaCl, 5.9 mM NaHCO_3_, 1.47 mM KCl, 0.31 mM NaHPO_4_, 1.05 mM MgCl_2_, 0.63 mM CaCl_2_, 4.99 mM Hepes, 22.7 mM glucose, pH 7.4). 

Washed erythrocytes: Citrated whole blood was centrifuged at 800 *g* for 10 min, and the pellet was carefully washed with Tris-Sucrose buffer (10 mM Tris/HCl, 250 mM Sucrose; pH 7.4) and centrifuged 2000 *g* for 5 min, three times. 

### 5.7. Platelet Aggregation Assay

Platelet aggregation in PRP and washed platelets were carried out as previously recorded [26]. Platelet aggregation was monitored in a Chrono-log aggregometer, model 490 (Chrono-Log Corporation, Havertown, PA, USA), at 37 °C with a bar stirring speed set to 1000 rpm. The dose of 0.3 µM of LgRec1, EGFP, and EGFP-LgRec1 was evaluated on platelet aggregation. The agonists ADP (final concentration, 10 µM) was used as positive control for platelet aggregation.

### 5.8. Determination of Hemolytic Activity

The tests were performed in six replicates, using 1 × 10^8^ red cells in 100 μL of diluted proteins (0.75; 0.375; 0.187 µM) per well in 96-well U-bottom plates, as follows: Indirect hemolytic activity: Washed erythrocytes were incubated with EGFP, LgRec1, or EGFP-LgRec1 (0.75, 0.375 and 0.187 µM, each) for 4 h at 37 °C. After that period, the erythrocytes were incubated with 50 μL of serum and the positive control incubated with 1% Triton X-100 for 1 h at 37 °C under agitation. Direct hemolytic activity: Washed erythrocytes were incubated with EGFP, LgRec1, or EGFP-LgRec1 (0.75, 0.375 and 0.187 µM, each) in the presence of 1 mM of Ca^2+^ for 12 h at 37 °C. After that period, the erythrocytes were incubated with 50 μL of Tris-Sucrose and the positive control was done as above. After the incubation, the plate was centrifuged at 2000 *g* for 5 min and the supernatant read at 415 nm. The values were converted to percentages considering the 100% the hemolysis caused by Triton X-100. For the tests, serum and red cells from the same donor were used to prevent complement activation by recognition of antibodies by the ABO blood system.

### 5.9. Confocal Immunofluorescence 

Washed platelets (4 × 10^8^ platelets) were fixated in microscope slides using the Fixative Solution (Methanol 100%) from the HEMA 3^®^ kit (Thermo Fisher Scientific Scientific, Waltham, MA, USA) and washed with water. The fixated platelets were incubated with 500 μL of LgRec1 plus EGFP, EGFP, or EGFP-LgRec1 (4 µM, each) in the microscope slides for 2 h at 37 °C, then washed 3 times with Tyrode’s solution. 

PRP (4 × 10^8^ platelets) were incubated in aggregation conditions with 500 μL of LgRec1 plus EGFP, EGFP, or EGFP-LgRec1 (4 µM, each) in tubes for 2 h at 37 °C, then washed 3 times with Tyrode’s solution. Then, the platelet aggregate was transferred to microscope slides. Samples were then observed using a confocal fluorescence microscope (Confocal Radiance 2100, BioRad, Hercules, CA, USA) coupled to a Nikon-Eclipse E800 with Plan-Apochromatic objective (Sciences and Technologies Group Instruments Division, Melville, NY, USA). 

### 5.10. Flow Cytometry 

Chimeric protein binding experiments were performed on a flow cytometer (BD FACSCalibur System, BD Biosciences, Franklin Lakes, NJ, USA). Washed platelets (4 × 10^8^ cells) were incubated with EGFP, LgRec1 plus EGFP or EGFP-LgRec1 (4 µM, each) for 2 h at 37 °C. Treated washed platelets were separated from debris based on the forward- and side-scatter profiles (data not shown). Staining positive was defined as those with a fluorescence intensity above that of the untreated platelets. The percentage of EGFP positive cells were calculated using FlowJo software version 7.5 (FlowJo LLC, Ashland, OR, USA). Data from 100,000 events were collected and analyzed. All samples were performed in duplicate and the results expressed as the percentage of EGFP positive cells calculated from untreated platelets.

### 5.11. Phosphatidylserine Exposure

To identify the exposure of phosphatidylserine (PS) annexin-V binding experiments were performed using the PE Annexin V Apoptosis Detection Kit I (BD Biosciences) in a flow cytometer (BD FACSCalibur System, BD Biosciences, Franklin Lakes, NJ, USA). Washed platelets (4 × 10^8^ cells) were incubated with EGFP, LgRec1 plus EGFP or EGFP-LgRec1 (4 µM, each) for 1 h and 45 min at 37 °C. PE-Annexin V was then added and incubated for 15 min at room temperature (25 °C) in the dark. Platelets were separated from debris based on the forward- and side-scatter profiles (data not shown). All samples were performed in duplicate (10^4^ events per data acquisition file). The data were analyzed using FlowJo software version 7.5 (FlowJo LLC, Ashland, OR, USA) and the results expressed as the percentage of PE-Annexin V positive cells calculated from untreated platelets.

### 5.12. Lactate Dehydrogenase Leakage

The release of cytosolic enzyme lactate dehydrogenase (LDH) to the supernatant of platelets was determined as a marker of cell membrane integrity. Washed platelets (4 × 10^8^ cells/mL) were incubated with EGFP, LgRec1 plus EGFP, or EGFP-LgRec1 (4 µM, each) for 15 min at 37 °C. Negative control and positive control cytolysis consisted of medium of untreated cells and medium from cells incubated with 0.1% (*v*/*v*) of Triton X-100, respectively. After incubation, cells were centrifuged at 10,000 *g* for 15 min. The supernatant was used to determine the leakage of lactate dehydrogenase (LDH) from platelets using the LDH Cytotoxicity Assay Kit (Cayman Chemical, Ann Arbor, MI, USA) following the manufacturer’s protocol. 

### 5.13. Statistical Analyses

Statistical analyses were performed using analysis of variance (ANOVA) with the posttest Bonferroni test in the GraphPad Prism 5 software version 5.01, 2007. (GraphPad Software, Inc., La Jolla, CA, USA). Significance was considered when *p* ≤ 0.05.
